# An Insightful Observation Leading to a Late Diagnosis of Spinal Muscular Atrophy: A Case Report

**DOI:** 10.7759/cureus.59786

**Published:** 2024-05-07

**Authors:** Felix Rivera Troia, Fernando Ocasio Villa

**Affiliations:** 1 Genetics, University of Medicine and Health Sciences, Basseterre, KNA; 2 Genetics, Western Oncology Cancer Center, Mayagüez Medical Center, Mayagüez, PRI

**Keywords:** late diagnosis, smn1 gene, survival motor neuron gene, neuromuscular diseases, spinal muscular atrophy (sma)

## Abstract

Spinal muscular atrophy (SMA) is a rare autosomal recessive neuromuscular disorder characterized by the loss of motor neurons in the spinal cord that results in progressive muscle weakness and atrophy. Most often, the gene involved in this disorder is the survival motor neuron (SMN1) gene, located on the telomeric regions of chromosome 5q13. This gene is involved in the processing of pre-mRNA required for the formation of dendrites and axons. Here we present the case of a 47-year-old female with an extensive past medical history of progressive muscle weakness who, after numerous specialist evaluations, was sent for germline mutation panel sequencing and analysis and was incidentally found to have a pathogenic heterozygous deletion encompassing the exon 8 region of the SMN1 gene. This case report aims to highlight the importance of timely identification and management for individuals who present with early clinical signs of the disease to reduce the morbidity and mortality associated with it.

## Introduction

Spinal muscular atrophy (SMA) is an autosomal recessive neuromuscular disorder. The disease has a prevalence of around 1 per 100,000 persons. It primarily affects the motor neurons of the anterior horn cells of the central nervous system [1]. These motor neurons are responsible for skeletal muscle activity in the body [2]. This, in turn, leads to progressive muscle weakness and wasting [3]. There are four types of SMA, and the classification of each is dependent on the age of the presentation. Type I SMA, also known as infantile-onset SMA or Werdnig-Hoffmann disease, is the most common type and is usually identified by six months of age. Type II SMA starts to manifest between the ages of six and 18 months. In Types III and IV, symptoms usually develop after 18 months or 21 years of age, respectively. Phenotypically speaking, the severity of the disease is inversely related to the age of onset; that is to say, the earlier the onset of symptoms, the more severe the disease course [4].

The most common form of SMA is due to a deletion mutation in exon 7 found in the survival motor neuron (SMN1) gene; this encompasses around 95% of most mutations [5]. The remaining 5% includes other mutations in different locations. This gene is located in the telomeric regions of chromosome 5q13 and is important for maintaining the integrity of motor neurons [1]. An identical gene known as SMN2 is hypothesized to be involved in the disease as well. The difference between both genes is that a single nucleotide variant (C→T) on exon 7 is present on SMN2. Through the process of RNA modification, SMN2 manufactures minimal amounts of functioning SMN1 protein, further contributing to disease severity at a genetic level. Although similar, this functionally deficient gene and its relation to SMN1 suggest that it is a potent disease modifier of SMA, which is why it is also a target for potential therapies [6].

As we understand, there are few cases reported of SMA being diagnosed this late in a person’s life, and we believe that highlighting the importance of clinically being able to identify the disease as early as possible may aid in modifying the progression of the disease course as well as reducing the morbidity and mortality associated with it.

## Case presentation

We presented the case of a 47-year-old female who had been referred to the oncology clinic for the management of anemia secondary to chronic kidney disease. Upon reviewing the patient’s medical records, it was evident that she had been seen by multiple specialists and had been treated for, as long as she could recall, with steroids for an unspecified type of muscular dystrophy. Further review of her past family history showed evidence of consanguinity in her parents as well. It was during one of her treatment sessions that one of the physicians in the clinic noticed her walking with an ataxic gait. The combination of this clinical finding and her family history of consanguinity prompted the physician to suggest she undergo a genetic panel test. Genetic sequencing and analysis was performed and showed that the patient possessed a pathogenic heterozygous deletion encompassing the exon 8 region (conventionally referred to as exon 7) of the SMN1 gene (Table 1), confirming a diagnosis of SMA.

**Table 1:** Genetic sequencing and analysis results for heterozygous deletion of the SMN1 inpatient and her offspring

	Gene	Variant	Zygosity	Variant classification
Patient	SMN1	Deletion (entire coding sequence)	Heterozygous	Pathogenic
	BIN1	c.961G>A (p.Gly321Arg)	Heterozygous	Uncertain significance
	PLEC	c.1081G>A (p.Ala361Thr)	Heterozygous	Uncertain significance
	RYR1	c.12407G>A (p.Arg4136His)	Heterozygous	Uncertain significance
Sibling 1	SMN1	Deletion (entire coding sequence)	Heterozygous	Pathogenic
	BIN1	c.961G>A (p.Gly321Arg)	Heterozygous	Uncertain significance
	RYR1	c.12407G>A (p.Arg4136His)	Heterozygous	Uncertain significance
Sibling 2	SMN1	Deletion (entire coding sequence)	Heterozygous	Pathogenic
	RYR1	c.12407G>A (p.Arg4136His)	Heterozygous	Uncertain significance

The patient was referred for genetic counseling, where she was informed about her condition and the risk of progression. Additionally, the patient’s offspring underwent testing and were found to be carriers of the same mutation present in their mother (Table 1), although they showed no clinical presentation of the disease. Currently, the patient is being informed about several trials and management options available.

## Discussion

In this case, we have successfully confirmed the diagnosis of a patient with an SMA that had remained undiagnosed for the better part of 40 years. Diagnosing this disease proves to be somewhat difficult due to its many nonspecific clinical manifestations, such as progressive muscle weakness, ataxic gait, and muscle loss, among others [7]. Inclusively, some case reports focus attention on the long delay seen from symptom onset to genetic diagnosis [8]. Hirayama et al. presented the case of a 69-year-old woman with progressive limb weakness lasting 50 years who was later diagnosed with SMA [9]. Unlike our case, she was diagnosed using a CT scan of several muscles involved, followed by genetic testing confirmation.

SMA is a neuromuscular disorder characterized by progressive and irreversible loss of anterior horn cells in the spinal cord that essentially results in muscle weakness and atrophy. The clinical manifestations of SMA are quite heterogeneous [10]. Furthermore, SMA can be classified into four types according to the age of the onset of symptoms. Type I is the most common and severe form of SMA. It is also known as Werdnig-Hoffmann disease or infantile-onset SMA. Children with this type of disease have very limited movement, often accompanied by an inability to sit without support, swallow, or feed. Symptoms are often evident by the age of six months. Many children with this type do not live past the age of two. Type II SMA is an intermediate form, with symptoms becoming noticeable between the ages of six and 18 months. Children with this type may sit without support to a certain degree but are usually unable to walk. Type III is also known as Kugelberg-Welander disease. Symptoms present after 18 months of age and most closely resemble muscular dystrophy. Early signs may present as difficulty walking, and children eventually will need a wheelchair. Type IV SMA is very rare. It presents with the onset of symptoms as a young adult and is characterized by mild motor impairment [4].

SMA is an autosomal recessive motor neuron disorder with an estimated incidence of 1 in 10,000 live births and a carrier frequency of 1 in 40 to 1 in 60 [11]. The disease is caused by a mutation in the SMN gene, most commonly a deletion mutation on exon 7 [5]. SMN is an essential housekeeping protein involved in multiple processes, including DNA replication and repair, transcription, pre-mRNA splicing, translation, macromolecular trafficking, stress granule formation, cell cycle regulation, signal transduction, and maintenance of cytoskeletal dynamics [12,13]. All patients with SMA have insufficient amounts of SMN, which is encoded by two homologous genes known as SMN1 and SMN2. The difference between both genes is that a single nucleotide variant (C→T) on exon 7 is present on SMN2. It is because of this variance that, during the process of RNA modification, SMN2 is only able to manufacture minimal amounts of functioning SMN1 protein [6]. There are two currently approved therapies for SMA based on the restoration of SMN2 exon 7 inclusion [12,14]. Nusinersen, better known as Spinraza, is one of these disease-modifying therapies. This drug is administered intrathecally and works as an antisense oligonucleotide by binding to specific intron sequences downstream of exon 7 on the SMN2 gene, potentiating it and further increasing the production of normal SMN protein. This treatment is indicated for both adult and pediatric patients diagnosed with SMA [15]. Another drug, risdiplam, known by its brand name Evrysdi, has a similar mechanism of action. The biggest difference between these is their route of administration; Spinraza is administered as an intrathecal injection, whereas Evrysdi offers the ease of oral bioavailability [16]. Zolgensma is another treatment option for SMA that has a different mechanism of action from the other two. This drug works by directly delivering a functional SMN protein inside a viral-derived vector. This protein replaces the function of SMN1 and enables the production of SMN protein, which further contributes to the preservation of essential muscle function [17]. Understanding the function and mechanism of this protein may further aid in highlighting future pathways for therapeutic intervention.

## Conclusions

We present the case of a 47-year-old female with a long-standing history of progressive muscle weakness who was diagnosed late in life with SMA. To our understanding, there are very few reports of late diagnoses of this disease, which makes this one special. SMA presentation can vary widely in onset and severity and often resembles other diseases, which commonly leads to misdiagnosis. Our goal in presenting this case is to emphasize the importance of being able to identify early in the course the clinical signs of this disease. Treatment of SMA requires a systematic understanding of its underlying pathophysiology. We urge clinicians to perform genetic testing when clinical suspicion points to SMA to ensure timely and accurate diagnosis as well as reduce the mortality and morbidity associated with the disease.
